# Differential roles of galanin on mechanical and cooling responses at the primary afferent nociceptor

**DOI:** 10.1186/1744-8069-8-41

**Published:** 2012-06-06

**Authors:** Richard P Hulse, Lucy F Donaldson, David Wynick

**Affiliations:** 1 School of Physiology and Pharmacology, University of Bristol, University Walk, Bristol, BS8 1TD, UK; 2 School of Clinical Sciences, University Walk, Lower Maudlin St, University Walk, Bristol, BS2 8AE, UK

**Keywords:** Galanin, Primary afferent, Cold, Neuropathic pain, Inflammatory pain

## Abstract

**Background:**

Galanin is expressed in a small percentage of intact small diameter sensory neurons of the dorsal root ganglia and in the afferent terminals of the superficial lamina of the dorsal horn of the spinal cord. The neuropeptide modulates nociception demonstrating dose-dependent pro- and anti-nociceptive actions in the naïve animal. Galanin also plays an important role in chronic pain, with the anti-nociceptive actions enhanced in rodent neuropathic pain models. In this study we compared the role played by galanin and its receptors in mechanical and cold allodynia by identifying individual rat C-fibre nociceptors and characterising their responses to mechanical or acetone stimulation.

**Results:**

Mechanically evoked responses in C-fibre nociceptors from naive rats were sensitised after close intra-arterial infusion of galanin or Gal2-11 (a galanin receptor-2/3 agonist) confirming previous data that galanin modulates nociception *via* activation of GalR2. In contrast, the same dose and route of administration of galanin, but not Gal2-11, inhibited acetone and menthol cooling evoked responses, demonstrating that this inhibitory mechanism is not mediated by activation of GalR2. We then used the partial saphenous nerve ligation injury model of neuropathic pain (PSNI) and the complete Freund’s adjuvant model of inflammation in the rat and demonstrated that close intra-arterial infusion of galanin, but not Gal2-11, reduced cooling evoked nociceptor activity and cooling allodynia in both paradigms, whilst galanin and Gal2-11 both decreased mechanical activation thresholds. A previously described transgenic mouse line which inducibly over-expresses galanin (Gal-OE) after nerve injury was then used to investigate whether manipulating the levels of endogenous galanin also modulates cooling evoked nociceptive behaviours after PSNI. Acetone withdrawal behaviours in naive mice showed no differences between Gal-OE and wildtype (WT) mice. 7-days after PSNI Gal-OE mice demonstrated a significant reduction in the duration of acetone-induced nociceptive behaviours compared to WT mice.

**Conclusions:**

These data identify a novel galaninergic mechanism that inhibits cooling evoked neuronal activity and nociceptive behaviours *via* a putative GalR1 mode of action that would also be consistent with a TRP channel-dependent mechanism.

## Background

Galanin is expressed at low levels in less than 5% of adult rodent dorsal root ganglia (DRG) neurons; those expressing galanin are predominantly small diameter C-fibre nociceptors. Higher levels of the neuropeptide are expressed in the primary afferent terminals in the superficial layers of the dorsal horn in naïve animals [1]. Levels of galanin in the rodent, primate and human DRG markedly rise after peripheral nerve injury [1,2]. Administration of galanin to either the peripheral [3] or central [4] nervous systems results in a significant alteration in mechanosensory nociceptive behaviours. These actions are dose-dependent with facilitation occurring at low concentrations and inhibition at higher concentrations [3,4]. After nerve injury, when a subset of mechano-nociceptors are sensitised to mechanical stimulation [5] and the endogenous levels of galanin in the DRG are high, there is good agreement in the literature that the anti-nociceptive actions of galanin are enhanced in rodent models of neuropathic pain [6]. In contrast to the extensive dataset on galanin modulation of mechanosensory behaviours, there have been few studies on galanin and cold behaviours. Work to date has shown that in the naïve uninjured animal intrathecal infusion of low dose galanin facilitates cold nociceptive behavioural responses [7]. In contrast, in a model of neuropathic pain galanin is anti-nociceptive, attenuating cold pain [8].

The nociceptive effects of galanin are mediated by the activation of one or more of three G-protein coupled galanin receptor subtypes, designated GalR1, GalR2 and GalR3. Studies using *in-situ* hybridization have shown that GalR1 and GalR2 mRNAs are expressed by 51% and 83% of adult rat DRG neurons respectively [9], and the levels of both sub-types decrease after nerve injury [10,11]. In contrast, no change in the expression of either subtype was observed in the dorsal horn of the spinal cord after axotomy [12]. Expression of GalR3 in the spinal cord and DRG in both rat and mouse is very low as determined by RT-70 PCR [13,14], and *in situ* hybridization [15]. We and others have shown that the central and peripheral anti-nociceptive effects of galanin on mechanosensory thresholds are principally mediated by activation of GalR1 [7] and GalR2 [3], respectively. Only two papers have studied which of the galanin receptors mediate the effects of galanin on cold nociception. Liu et al. showed that intrathecal infusion of low dose Gal2-11 (a GalR2/3-specific agonist) increased nociceptive responses to acetone in the naive rat, similar to that seen with galanin [7]. In contrast, Blakeman et al. demonstrated an increase in cold pain scores in naive GalR1-KO mice compared to strain-matched wild-type (WT) controls [16].

Here we investigated the role that galanin and its receptors play at the primary afferent with particular reference to cold sensitivity, demonstrating that close intra-arterial administration of galanin inhibits C-fibre nociceptor cooling activity in normal, nerve-injured and inflamed animals. These actions appear to be independent of GalR2 activation. These data identify a novel galaninergic mechanism that inhibits cooling-evoked neuronal activity and nociceptive behaviours consistent with a TRP channel-dependent mechanism.

## Results

Using multi-unit recordings in naive rats and models of neuropathic and inflammatory pain, we studied the differential effects of galanin and the GalR2/3-specific agonist Gal2-11 on primary afferent nociceptor activities to mechanical and cooling stimulation.

### Differential effects of galanin and Gal2-11 on naïve primary afferent nociceptor activities to mechanical and cooling stimulation

**Figure 1:**
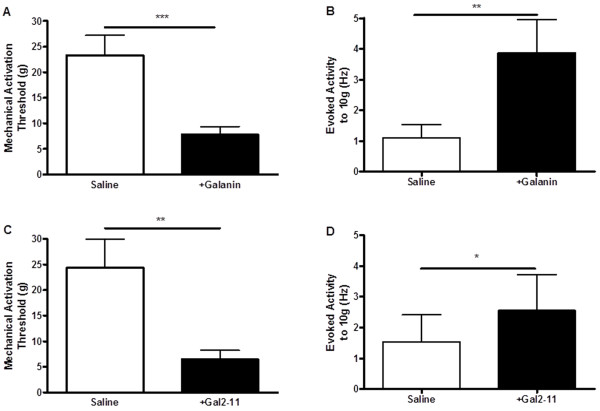
**Facilitatory actions of galanin*via*activation of GalR2 on mechano-nociceptor afferents [A]** Mechanical activation thresholds were reduced by c.i.a 100 μM galanin in naïve rats (*** p < 0.001 Mann Whitney test, n = 24) and **[B]** mechanically evoked activity was increased by c.i.a 100 μM galanin in response to a 10 g vF hair mechanical stimulus (**p < 0.01 paired *t*-test). **[C]** Mechanical activation thresholds were reduced following c.i.a 100 μM Gal2-11 administration (** p < 0.01 Mann Whitney test, n = 14) and **[D]** mechanically evoked activity was increased by c.i.a 100 μM Gal2-11 in response to a 10 g vF hair mechanical stimulus (* p < 0.01 paired *t*-test, n = 14).

Galanin 100 μM was administered *via* c.i.a. (close intra-arterial) as previously described [17]. Mechanical activation thresholds were significantly reduced following galanin administration (Figure 1A, ***p < 0.001) and mechanically evoked activity was enhanced (Figure 1B, ** p < 0.01). Similarly, c.i.a. administration of 100 μM Gal2-11 led to a significant reduction in activation thresholds of mechanically sensitive C-fibre afferents (Figure 1C, **p < 0.01) and an increase in mechanically evoked activity (Figure 1D, *p < 0.05).

**Figure 2:**
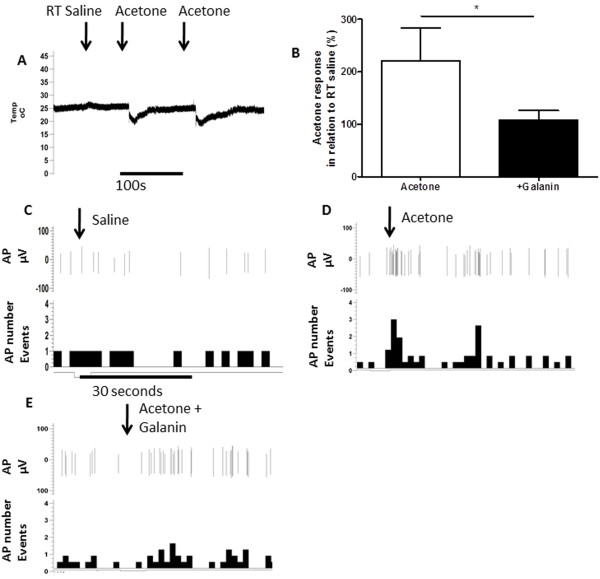
**Exogenous galanin attenuates cooling responses. [A]** Digitised trace of recording of subcutaneous skin temperature of the dorsal surface of the rat hindpaw. Room temperature saline did not alter skin temperature. Application of acetone led to a reduction in skin temperature. This returned to normal within 100 seconds. A second application of acetone again resulted in a similar drop in temperature. **[B]** Acetone application to the receptive field of an identified cooling sensitive primary afferent led to a significant increase in neuronal firing, which was significantly greater than the control application of room temperature saline (**p < 0.01 one way ANOVA with post-hoc Bonferroni test, n = 6). Cooling evoked activity was significantly inhibited following administration of c.i.a. 100 μM galanin (*p < 0.05 Paired *t*-test, n = 6). **[C]** Example trace of an acetone/cooling sensitive C fibre in response to room temperature saline. **[D]** Example trace of an acetone/cooling sensitive C fibre. **[E]** Acetone evoked activity was attenuated in the same C fibre by c.i.a. 100 μM galanin. AP = action potential, AP number events = total number of action potentials occurring per second.

**Figure 3:**
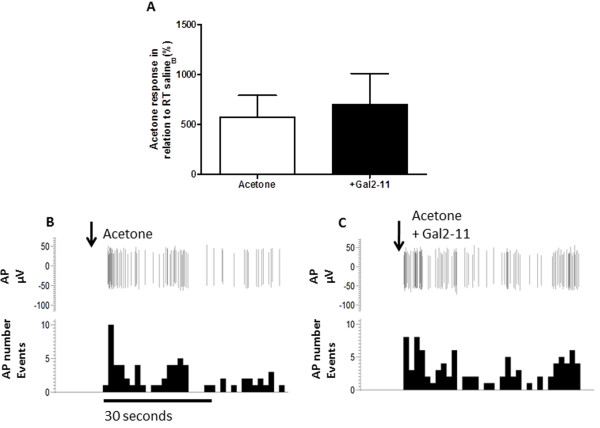
**Acetone evoked activity is not inhibited by GalR2/3 activation. [A]** c.i.a. 100 μM Gal2-11 did not alter acetone-induced cooling responses (n = 6). **[B]** Example trace of an acetone/cooling sensitive C fibre. **[C]** The acetone evoked activity was not altered by Gal2-11. AP = action potential, AP number events = total number of action potentials occurring per second.

Application of acetone, which leads to mild nociceptive behaviours in naïve rodents [18], reduced skin temperature for approximately 30–60 seconds by ~5°C from a resting temperature of ~25°C (Figure 2A). Room temperature saline was used to confirm that the mechanical application of a drop of fluid to the receptive field did not alter skin temperature (Figure 2A) or neuronal responses (Figure 2B). On-going neuronal activity in cooling primary afferents (as previously described [19]), was identified from the application of room temperature saline (Figures 2B and 3A). All cooling sensitive afferents had CVs less than 1 m/s and were thus classified as cooling sensitive C-fibres. Application of acetone to the receptive field significantly increased afferent evoked activity compared to room temperature saline (Figures 2B &3A *p < 0.05, **p < 0.01 one way ANOVA). Acetone responses following c.i.a. 100 μM galanin were significantly reduced (Figures 2B &2D, * p < 0.05 Paired *t*-test). In contrast, 100 μM Gal2-11 had no effect on acetone evoked responses (Figures 3A and 3C).

**Figure 4:**
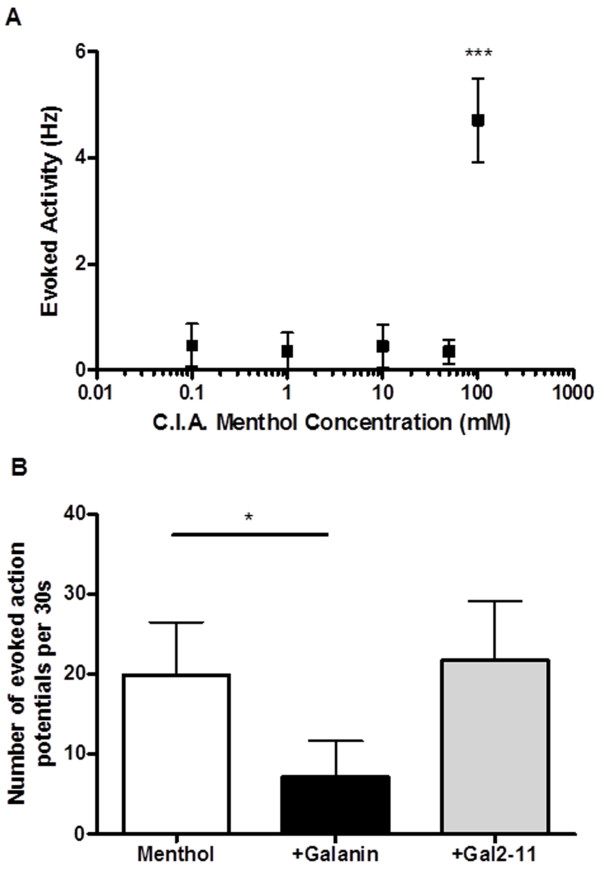
**Menthol evoked activity is not inhibited by GalR2/3 activation. [A]** The concentration response relationship demonstrates that acetone sensitive C fibres responds only to c.i.a. 100 mM menthol (***p < 0.001, one way ANOVA with post-hoc Bonferroni test, n = 4). **[B]** Menthol evoked activity was reduced by c.i.a. 100 μM galanin but not by c.i.a. 100 μM Gal2-11 (* p < 0.05, one way ANOVA with post-hoc Bonferroni test, n = 7).

We then investigated the effects of galanin and Gal2-11 on the neuronal activity elicited after application of the cooling agent menthol; an activator of the cold sensitive channel TRPM8 [20]. Menthol evoked action potentials in 8 out of 10 acetone/cooling sensitive afferents. A single c.i.a bolus dose of 100 mM menthol led to a robust increase in evoked activity (Figure 4A) which was significantly reduced when preceded by c.i.a administration of 100 μM galanin (Figure 4B, *p < 0.05 one way ANOVA) but not by c.i.a 100 μM Gal2-11 (Figure 4B).

### Differential effects of galanin and Gal2-11 on primary afferent nociceptor activities to mechanical and cooling stimulation in chronic pain models

**Figure 5:**
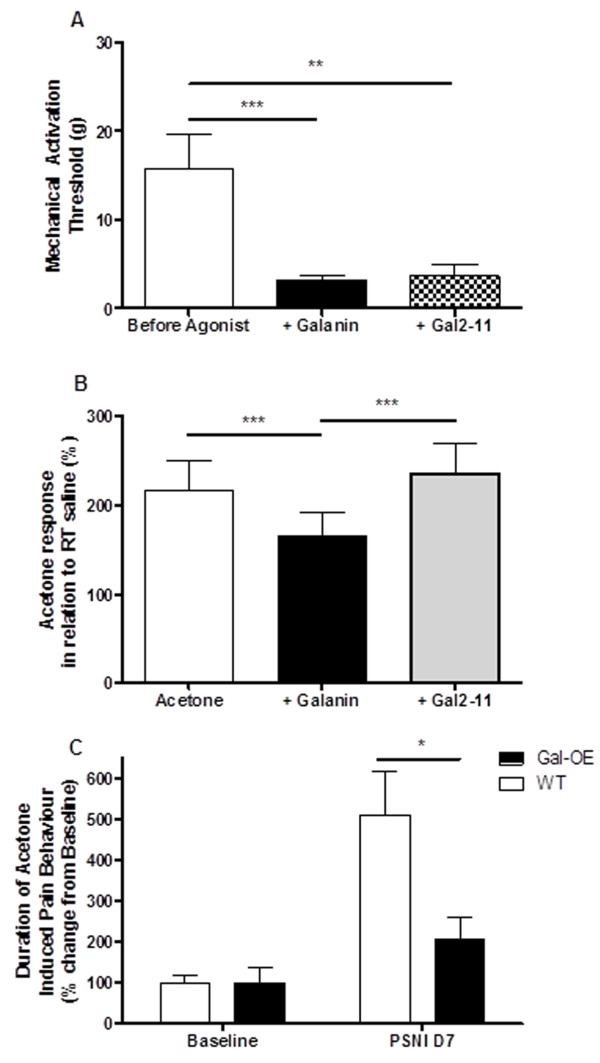
**Differential role of galanin in a model of neuropathic pain [A]** Exogenous c.i.a 100 μM galanin and c.i.a 100 μM Gal2-11 both facilitated mechano-nociceptor responses in the partial saphenous nerve injury model of neuropathic pain (** p < 0.01, *** p < 0.001, Kruskal Wallis test with Dunn’s multiple comparison test, galanin n = 15, Gal2-11 n = 6). **[B]** In the PSNI neuropathic pain model acetone application to the receptive field of cold sensitive C fibres led to a significant increase in evoked activity (***p < 0.001, Kruskal-Wallis with Dunn’s multiple comparison test). Acetone-induced evoked activity was inhibited by c.i.a 100 μM galanin but was not altered by c.i.a 100 μM Gal2-11 (***p < 0.001, one way ANOVA with post-hoc Bonferroni test, n = 10) **[C]** Acetone-induced withdrawal behaviours in naïve Gal-OE transgenic mice did not differ from WT controls. 7-days after nerve injury the Gal-OE mice demonstrated a significant reduction in cooling pain like behaviours compared to matched WT mice (*p < 0.01, two way ANOVA with post-hoc Bonferroni test, n = 7).

In rats that had undergone PSNI that results in mechanical and cold allodynia, c.i.a. 100 μM galanin and c.i.a 100 μM Gal2-11 both led to a significant reduction in mechanical activation thresholds of C-fibres (Figure 5A, ***p < 0.001). In contrast, acetone-evoked activity in C-fibre afferents was significantly attenuated following c.i.a 100 μM galanin (Figure 5B, *p < 0.05, ***p < 0.001) but not by c.i.a 100 μM Gal2-11 (Figure 5B).

A previously described transgenic mouse line which inducibly over-expresses galanin (Gal-OE) after nerve injury [21] was used to investigate whether manipulating the levels of endogenous galanin also modulates cooling evoked nociceptive behaviours after PSNI. We have previously shown a marked attenuation of mechanical allodynia in these mice after PSNI [3,21]. Acetone withdrawal behaviours in naive mice showed no differences between Gal-OE and WT mice. 7-days after PSNI Gal-OE mice demonstrated a significant reduction in the duration of acetone-induced nociceptive behaviours compared to WT mice (Figure 5C, *p < 0.01 two way ANOVA).

**Figure 6:**
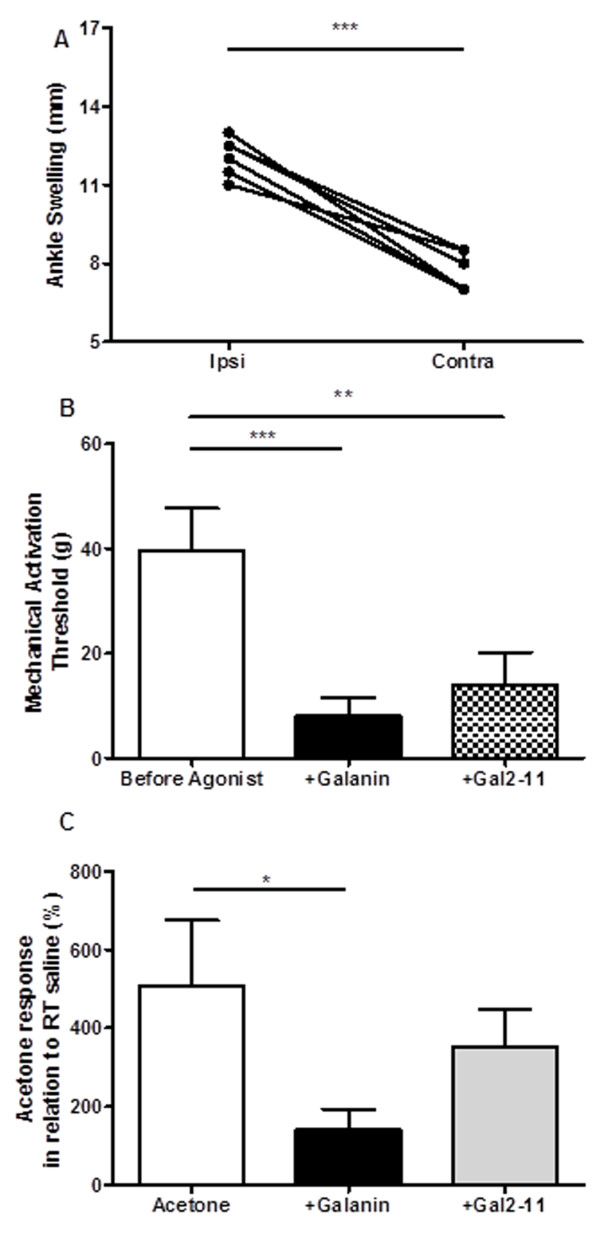
**Differential role of galanin in CFA inflammation [A]** CFA inflammation was confirmed through significant ipsilateral ankle joint swelling compared to the contralateral joint (***p < 0.001, paired *t*-test, n = 6). **[B]** Exogenous c.i.a. 100 μM galanin and c.i.a. 100 μM Gal2-11 both significantly facilitated mechano-nociceptor mechanical responses in CFA inflamed rats (** p < 0.01, *** p < 0.001, Kruskal-Wallis with Dunns multiple comparison test, n = 9). **[C]** Exogenous c.i.a. 100 μM galanin, but not c.i.a. 100 μM Gal2-11, reduced acetone evoked activity (* p < 0.05, one way ANOVA with post-hoc Bonferroni, n = 6).

Similar responses were also observed following CFA treatment in the rat. The degree of inflammation at 5 days was confirmed by measuring ankle joint swelling (Figure 6A, ***p < 0.001, paired *t*-test). 100 μM galanin and 100 μM Gal2-11 by c.i.a administration both led to a significant reduction in mechanical activation thresholds of mechanically sensitive C-fibres (Figure 6B, **p < 0.01, ***p < 0.001). In contrast, c.i.a 100 μM galanin, but not c.i.a 100 μM Gal2-11, attenuated the acetone evoked responses in cooling sensitive C-fibres (Figure 6C, *p < 0.05 one way ANOVA).

## Discussion

This study describes the differential roles played by galanin in the peripheral nervous system with respect to mechanical- and cold-pain behaviours in the naive state and following models of neuropathic and inflammatory pain. Here we show opposing actions of galanin, with inhibition of cold- and facilitation of mechanical-responses, in C-fibre nociceptors.

Cold allodynia (the sensation of pain to a normally innocuous stimulus) is a common complaint of patients with neuropathic pain, irrespective of the underlying cause [22-24] and similar findings are found in a range of rodent models of neuropathic and inflammatory pain behaviours following stimulation with acetone or menthol [25-28]. Cooling detection in humans is highly variable. It occurs after a drop in temperature of only a few degrees [29,30] and is highly correlated with the activation temperature of TRPM8 [31-33] (31-27°C). Cold pain thresholds are also highly variable, but are usually reported at lower temperatures [31,34] (<10°C). Patients with neuropathic pain report pain after only a small reduction in skin temperature with thresholds reported to be >20°C [35]. At these temperatures, symptoms are described as painful with intense pain occurring at lower temperatures [23] (~15°C). Recent work has made considerable progress in defining the molecular transducers of cutaneous thermo-sensation and the mechanisms that underlie cold allodynia. A number of thermal sensors have been identified which all belong to the Transient Receptor Potential (TRP) ion channel family. The best characterised cold receptor is TRPM8 [36-38]. TRPA1 was proposed as a noxious cold sensor but more recent evidence has disputed a specific contribution for TRPA1 in cold transduction [28,39,40]. TRPM8 is expressed by a subpopulation of small diameter C-fibre neurons [41]. TRPM8 responds to cooling agents such as acetone with an activation temperature threshold of ~26°C, with activity increasing in magnitude down to 8°C [36,38]. Menthol activates TRPM8 [42] and does not influence TRPA1 activity in non-human mammals [20,43,44]. Consistent with these findings, behavioural nociceptive responses over a range of innocuous and noxious temperatures and to the application of menthol are attenuated in TRPM8-KO mice, whereas these responses remain intact in the TRPA1-KO [39].

In the present study we have shown that galanin, but not Gal2-11, inhibits acetone and menthol responses in the naive rodent and following models of neuropathic and inflammatory pain. Similarly, overexpression of galanin after nerve injury in the DRG of Gal-OE mice led to the inhibition of cooling pain responses compared to those of wild type mice, confirming that high levels of endogenous galanin also inhibit cooling behaviours. The modulatory effects of galanin on cooling are independent of GalR2 and GalR3 activation since Gal2-11, which is equally selective for GalR2 and GalR3 [45], does not inhibit cooling pain behaviours. Further, since GalR3 is expressed at very low levels in the primary nociceptor in the intact and injured state [14], these findings imply the effects of galanin on cold responses are mediated by activation of GalR1. These findings are consistent with our work on the Gal-KO [46] and that of Blakeman et al. which demonstrate an increase in cold pain scores in naive GalR1-KO mice compared to strain-matched wild-type (WT) controls [16].

Since galanin suppresses the effects of acetone and menthol and the majority of acetone-sensitive C-fibres were also activated by c.i.a administration of menthol, this implies a mechanism *via* the modulation of the TRPM8 cold sensor. To date, no publication has investigated the effects of galanin and its receptors on TRPM8 activity. However, peripheral administration of galanin has been shown to modulate TRPV1 (Transient Receptor Potential Vanilloid) activity following inflammation, as GalR1-activation inhibits capsaicin-evoked pain behaviours [47], whilst GalR2-activation facilitates the same nociceptive responses [48]. Of note, all three galanin receptors couple to G_i/o_ and inhibit adenylyl cyclase [49,50], whilst GalR2 also signals *via* G_q/11_ to activate phospholipase C (PLC) and protein kinase C (PKC) [51]. Previous work has shown that TRPM8 activity in the DRG is inhibited by the G_i/o_ adenylyl cyclase pathway [52], which is again consistent with a GalR1-dependent modulation of the channel.

In contrast to the above inhibitory effects of peripheral administration of galanin on cooling nociception, here we show that c.i.a administration of the same dose (100 μM) of galanin and Gal2-11 both facilitate mechano-sensory responses. The nature and magnitude of these changes (Figure 1) are very similar to our previously published dataset using injection of 100nM galanin or 100nM Gal2-11 directly into the afferent receptive field in naïve adult male rats [3] (Figures 15A and 6B in that paper). This implies that the local concentration of galanin or Gal2-11 at the nociceptor is ~1000-fold lower than that administered by c.i.a bolus injection which may be explained by a combination of dilution of the peptides by the blood volume of the hind limb and only partial tissue penetration of the peptides. Our current and previous findings confirm that the peripheral mechano-nociceptive effects of galanin appear to be predominantly dependent on activation of GalR2, in contrast to the effects of galanin administered intrathecally which appear to be mediated by activation of GalR1 [7].

In conclusion, peripheral administration of galanin, but not Gal2-11, inhibits acetone- and menthol-evoked activity in C-fibre nociceptors in the normal animal and in models of neuropathic and inflammatory pain. These data identify a novel putative GalR1-dependent pathway that inhibits cooling-evoked neuronal activity and nociceptive behaviours, consistent with a TRPM8-dependent mechanism. Taken together with the known peripheral GalR2-dependent modulation of mechano-nociception, these findings imply that activation of peripheral GalR1 and GalR2 will both be important to treat cold and mechanical allodynia that occurs in various neuropathic pain conditions.

## Materials and methods

### Animals

Experiments were performed on adult male Wistar rats (250 g-350 g) and mice (~25 g). 47 Wistar rats were used for electrophysiological experiments. 10 transgenic mice that inducibly over-express galanin in the DRG as previously described [3], and 10 matched WT controls were used in nociceptive behavioural testing. Animals were fed standard chow and water *ad libitum* and all experiments were carried out in accordance with the United Kingdom Animals (Scientific Procedures) Act 1986.

### Electrophysiological recordings

Anaesthesia was induced using sodium pentobarbital (60 mg/kg i.p. rats, Sigma-Aldrich, UK) and were maintained deeply anaesthetised and areflexive for the duration of the experiment (~20 mg/kg/hr, sodium pentobarbital delivered intravenously *via* cannulation of the external jugular vein). A tracheotomy was performed to maintain the airway. Monitoring of blood pressure was carried out *via* a femoral artery cannulation of the left hind limb. Rodent body temperature was maintained at ~37.5°C by means of a feedback controlled heater and rectal thermistor. At the end of all experiments, rats were overdosed with a single large bolus of (60 mg/ml) sodium pentobarbital.

An incision was made along the inguinal fossa of the right hind leg to expose the saphenous nerve which runs superficially under the skin adjacent to the femoral vasculature. Using blunt dissection, connective tissue was removed and the skin was freed. Using the skin a pool was formed by attaching the skin to a metal pool ring, which was filled with warmed mineral oil (Sigma-Aldrich, UK). Fine forceps were used to dissect out a sufficient length of the nerve that was placed onto a dental mirror. The main saphenous nerve trunk was further prepared by removing the surrounding epineurium. Fine filaments were dissected out and cut proximally. These nerve filaments were placed onto bipolar platinum recording electrodes to record from individual afferent responses. Identified fine nerve filaments were ensured to have a low number of responsive receptive fields in the hindpaw (<3), with these receptive fields not overlapping. The neuronal activity of individual afferents was captured and spike sorted offline using micro1401 and Spike2 software (C.E.D. Cambridge, UK).

### Afferent characterisation

Primary afferents were identified using search stimuli of both blunt/pinch mechanical stimulation of the medial surface of the hindpaw, and electrical stimulation. Each afferent identified was characterised with the same protocol as described below. Upon identification of a receptive field the conduction velocity (CV) for the afferent was recorded. Electrical stimulation of the receptive field (0.5 ms stimulus duration, up to 100 V, every 3 seconds) was used to calculate the conduction velocity of the afferent. Three or more robust CVs from the receptive field were required to confirm reproducibility and confirmation of afferent excitation. CVs less than 1 m/s were classed as C fibres, those with greater than 1 m/s as A fibres [53]. On-going neuronal activity was monitored for 100 seconds following CV characterisation. This 100 second period recorded any neuronal activity from the afferent that occurred without any stimulus being applied to the receptive field. Mechanical responses were recorded using calibrated plastic filaments; von Frey hairs (Linton Instruments, UK) and brush stimulation. Von Frey hairs (vF) were used to elicit mechanical responses, with each vF hair applied three times for a maximum of five seconds. The mechanical activation threshold was determined using an adapted up and down method, and the lowest vF hair that elicited a robust response (response >3 action potentials) was noted as the activation threshold [54]. Afferents were also tested for innocuous cooling responses. Acetone was applied to the receptive field to test for cooling responses. This stimulus leads to withdrawal behaviours [55] and is a standard tool to excite cooling neuronal responses [56,57]. Prior to acetone, a single drop of room temperature saline was applied (using a Gilson and 1 ml pipette tip) to the receptive field to identify any mechanical response occurring due to the application of a drop of fluid. Neuronal activity was recorded for 30 seconds post stimulation. Room temperature saline and acetone were each applied a total of 3 times with 5 minutes between each application to allow skin temperature and neuronal activity to recover to baseline levels. Mean acetone and saline evoked responses were calculated. To note, cooling afferent groups studied with drug administration include both CCs (cold sensitive C fibres) and CMCs (mechano-cold sensitive C fibres) as previously described [58,59]. Drug responses were not different between these two groups.

### Experimental protocol and drug administration

Following baseline mechanical and cooling characterisation of primary afferent fibres, drugs were administered *via* the close intra-arterial (c.i.a) route as previously described [17,60]. The left femoral artery was cannulated in the groin and the cannula was advanced proximally, approximately ~2.5 cm, towards the bifurcation of the descending aorta. Cannula positioning was confirmed by visual inspection upon termination of experiment. Menthol (Sigma-Aldrich), 100 μM galanin (Bachem) or 100 μM Gal2-11 (Sigma-Aldrich, UK), were made up in saline and injected and washed through the c.i.a. cannula with 400ul of saline (containing heparin 50units/ml). 5 minutes after galanin or Gal2-11 injection either mechanical, acetone and menthol responses were tested. Since galanin and Gal2-11 have short half-life of <10 minutes [46,61], in some experiments galanin and Gal2-11 were tested sequentially in the same animal to reduce the number of animals used. 30 minutes was allowed between peptides to allow for metabolism and removal. 100 mM menthol (Sigma-Aldrich, UK) was administered *via* c.i.a cannula as previously described [28,55,60]. Menthol was dissolved in 10% ethanol, 10% Tween80 and 80% saline and vehicle alone had no effect on neuronal activity (data not shown).

### Temperature recordings

A thermocouple was advanced under the skin of the medial surface of the hindpaw using a 25-gauge needle. The thermocouple was used to identify changes in skin temperature following application of various stimuli. Saline and acetone were applied and temperature changes monitored and recorded using a CED micro 1401 and Spike2 software as described previously [56].

### Chronic pain models

For all recovery surgeries; partial saphenous nerve ligation injury (PSNI) and complete Freund’s adjuvant (CFA), anaesthesia was induced using 4% halothane in oxygen and maintained with 2-3% for all procedures.

### PSNI surgery

The PSNI surgery was performed as previously described [57,62] on 19 male wistar rats and 20 male mice, leading to the characteristic neuropathic pain phenotypes of mechanical allodynia and cooling allodynia [57,62,63]. An incision was made along the inguinal fossa of the right hind leg and the saphenous nerve was isolated using blunt dissection. A sterile silk suture (mouse: 7.0 suture and rat: 4.0 suture) was used to tightly ligate 50% of the saphenous nerve. The skin incision was closed using size 4.0 sutures. The animals were allowed to recover and monitored for the duration of the experiment. Electrophysiological experimentation was performed 7 days after PSNI surgery.

### Complete freunds adjuvant (CFA) inflammation

Two 50 μl injections of CFA (1 mg/ml, Sigma-Aldrich, UK), one on either side of the tibio-tarsal ankle joint, were used to induce inflammation in nine male Wistar rats as previously described [60] for electrophysiological experimentation and left to develop for five days. This intervention leads to significant swelling of the ankle and hindpaw [64].

### Animal behaviour

Mice were habituated to behavioural testing enclosures (mesh floored holding chambers) the day prior to testing for 15 minutes. For testing days animals were placed into behavioural enclosures 10 minutes prior to testing. Using a 1 ml syringe, acetone was applied to the plantar surface of the hindpaw. The duration of flinching/pain like behaviour (seconds) was recorded immediately following acetone application for a total period of five minutes. This was performed three times for each hindpaw, from which the mean was calculated. Stimulations of the right and left hindpaw were alternated to prevent sensitisation and allow recovery of skin temperature. Baselines were taken for two separate recording sessions and were then used to generate the baseline timepoint. Seven days post nerve injury animals were re-tested for acetone responses.

### Statistical analysis

Paired *t*-test and one way ANOVA with post-hoc Bonferroni tests were used to determine primary afferent evoked activity to mechanical or cooling stimulation before and after drug, as appropriate (galanin, Gal2-11 and menthol, *p < 0.05, **p < 0.01, ***p < 0.001). Mechanical activation threshold was determined by Mann Whitney Test or Kruskal-Wallis test with Dunns multiple comparison test (*p < 0.05, **p < 0.01, ***p < 0.001). To determine if galanin or Gal2-11 facilitated mechanical responses (increased number of action potentials) to an evoked stimulus, a cut off was set as two standard deviations above and below the mean of the evoked activity. In addition, cooling sensitive primary afferent evoked activity in response to acetone was calculated through a fold change in activity compared to that following room temperature saline application. Percentage change of cold induced pain like behavioural responses following PSNI were calculated from baseline naïve animal responses. Two way ANOVA with post bonferroni tests was used to determine differences in the Gal-OE cooling behaviour analysis. All data are presented as mean ± SEM.

## Abbreviations

CFA: Complete Freunds adjuvant; c.i.a: Close intra arterial; DRG: Dorsal root ganglia; Gal: Galanin; GalR: Galanin receptor; Gal-OE: Galanin over-expressing transgenic mice; i.t: Intrathecal; KO: Knockout; PSNI: Partial saphenous nerve injury; WT: Wild-type mice.

## Competing interest

The authors declare no competing interest.

## Authors’ contributions

RH formulated the hypothesis, initiated and organized the study. DW and LD provided the funding. RH performed the experimental work and analysed the data. RH, LD and DW drafted the manuscript. All authors read and approved the final manuscript.
